# Meiotic segregation and post-meiotic drive of the *Festuca pratensis* B chromosome

**DOI:** 10.1007/s10577-023-09728-6

**Published:** 2023-09-02

**Authors:** Rahman Ebrahimzadegan, Jörg Fuchs, Jianyong Chen, Veit Schubert, Armin Meister, Andreas Houben, Ghader Mirzaghaderi

**Affiliations:** 1 Department of Plant Production and Genetics, Faculty of Agriculture, University of Kurdistan, Sanandaj, 66177-15175 Iran; 2 Leibniz Institute of Plant Genetics and Crop Plant Research (IPK) Gatersleben, 06466 Seeland, Germany

**Keywords:** B chromosome, meiosis, pollen mitosis, chromosome drive mechanism, CENH3, nondisjunction

## Abstract

In many species, the transmission of B chromosomes (Bs) does not follow the Mendelian laws of equal segregation and independent assortment. This deviation results in transmission rates of Bs higher than 0.5, a process known as “chromosome drive”. Here, we studied the behavior of the 103 Mbp-large B chromosome of *Festuca pratensis* during all meiotic and mitotic stages of microsporogenesis. Mostly, the B chromosome of *F*. *pratensis* segregates during meiosis like standard A chromosomes (As). In some cases, the B passes through meiosis in a non-Mendelian segregation leading to their accumulation already in meiosis. However, a true drive of the B happens during the first pollen mitosis, by which the B preferentially migrates to the generative nucleus. During second pollen mitosis, B divides equally between the two sperms. Despite some differences in the frequency of drive between individuals with different numbers of Bs, at least 82% of drive was observed. Flow cytometry-based quantification of B-containing sperm nuclei agrees with the FISH data.

**Supplementary Information:**

The online version contains supplementary material available at 10.1007/s10577-023-09728-6.

## Introduction

Supernumerary B chromosomes (Bs) exist in thousands of species across the tree of life. They are found in some, but not all individuals within a population and can vary in number between individuals. In some species, Bs can even exceed the number of standard A chromosomes (As) (Jones and Rees 1982). Bs are usually present in all tissues of an organism carrying them, but in some plant species, they are absent in roots and only exist in the aerial tissue of the same individual (Mendelson and Zohary 1972; Ruban et al. 2020). Bs are not required for the normal growth and development of organisms possessing them (Houben 2017; Jones 1991). Because most Bs do not confer any benefits, they are thought to be parasitic elements that persist in populations by using the cellular machinery required for the inheritance and maintenance of A chromosomes (Beukeboom 1994; Östergren 1945). In addition to the adverse effects of Bs on the host (Nur et al. 1988; Randolph 1941), examples exist of their beneficial roles on the host, such as resistance to crown rust in *Avena sativa* (Dherawattana and Sadanaga 1973), induction of heat tolerance during early stages of male sporogenesis in rye (Pereira et al. 2017), positive effects on plant height and length and weight of grain in rice (Cheng et al. 2000), contribution in adaptive evolution as in the case of blast fungus *Magnaporthe oryzae* (Langner et al. 2021), and the increase of the plant survival rate under stress conditions (Holmes and Bougourd 1991).

In many species, the transmission of Bs does not follow the Mendelian laws of equal segregation and independent assortment. This deviation results in transmission rates of Bs higher than 0.5, a process known as “chromosome drive” (Camacho 2022; Chen et al. 2022; Houben 2017; Jones 1991). Depending on the species, drive occurs before meiosis, during meiosis, or post-meiotic divisions. The balance between the B chromosome drive and its adverse effects on the fertility and vigor of the host determines the maximum number of Bs tolerated by the host species (Bougourd and Jones 1997).

Nondisjunction of sister chromatids is a key component of the B chromosome drive process in many species (Banaei-Moghaddam et al. 2012; Chen et al. 2022; Houben 2017; Jones 2018). Nondisjunction occurs when sister chromatids are held together post-replication by DNA-DNA topological entanglements and unresolved sister chromatid cohesion. In rye and *Aegilops speltoides,* nondisjunction of B sister chromatids occurs during the first pollen mitosis. Nondisjoined B sister chromatids will be included in the generative nucleus or remain lagging; consequently, most vegetative nuclei receive only A chromosomes at the end of the first pollen grain mitosis (Banaei-Moghaddam et al. 2012; Wu et al. 2019). In addition to drive, the inheritance of Bs could also be influenced by mitotic and meiotic instability (Chen et al. 2022).

*Festuca pratensis* Huds. (meadow fescue) is a valuable perennial grass mainly used worldwide for forage, turf, and soil stabilization. In this diploid species, in addition to seven pairs of As, individuals with one to five Bs have been reported (Bosemark 1950; Bosemark 1954). Comparative repeat analysis of *F*. *pratensis* individuals with and without Bs revealed a set of A- and B-specific repeats. Applying a B-specific repeat as a FISH probe showed that the number of Bs is the same in roots and leaves within this species (Ebrahimzadegan et al. 2019). Classical analysis has shown that the B of *F*. *pratensis* shows drive caused by nondisjunction of sister chromatids during the first pollen mitosis (Bosemark 1954). This finding was based on reciprocal crosses in different cross combinations and microscopic observation of aceto-carmine–stained mitotic pollen grains.

To provide a deeper insight into the drive process of the *F*. *pratensis* B chromosome, we used FISH to trace the B during male gametogenesis. Application of flow cytometry allowed the quantification of Bs inside vegetative and sperm nuclei. Overall, depending on the number of Bs, we identified different ways of Bs segregation during meiosis, and their preferential accumulation in sperm nuclei during the first pollen grain mitosis.

## Materials and methods

### Plant material and growth conditions

Seeds of *Festuca pratensis* Huds. (meadow fescue) were obtained from the Research Institute of Forests and Rangelands, Tehran (Iran). *F*. *pratensis* requires 3 months of vernalization to initiate flowering (Ergon et al. 2016; Heide 1988). Therefore, plants were cultivated in autumn under field conditions at the University of Kurdistan, Sanandaj, Iran, and under controlled greenhouse conditions at the Leibniz Institute of Plant Genetics and Crop Plant Research (IPK) Gatersleben, Germany. For the latter, seedlings were cultivated at 4 °C for 3 months and then transferred to a greenhouse with 12-h dark (12 °C) and 12-h light (13 °C) for two months, followed by moving the plants to 16 °C with the same day-length for flowering.

### Screening of individuals harboring B chromosomes

Two different methods were used to identify B chromosome-containing individuals. Either the number of mitotic metaphase chromosomes was determined, or interphase nuclei were analyzed after hybridization of the B-specific probe Fp-Sat253, according to Ebrahimzadegan et al. (2019), with some changes in nuclei preparation. Briefly, leaf tissue was fixed in 3:1 ethanol:glacial acetic acid (v/v) for 1 h. For each individual, a 1-cm^2^ leaf section was chopped thoroughly in nuclei isolation buffer (15 mM Tris, 2 mM Na_2_EDTA, 0.5 mM spermine tetrahydrochloride, 80 mM KCl, 20 mM NaCl, 15 mM β-mercaptoethanol, 0.1% (v/v) Triton X-100; pH 7.5 (Doležel et al. 1998)) to prepare a cell suspension. In total, 100 μl of cell suspension was filtered through a 50 μm CellTrics filter (Sysmex-Partec, Germany) and spun for 5 min at 700 rpm per slide using the Shandon Cytospin 3. Slides were kept in 70% ethanol at −20 °C until FISH.

### Probe preparation

The A-specific Fp-Sat2 and B-specific Fp-Sat253 probes were prepared according to Ebrahimzadegan et al. (2019). Clone pTa71 containing a 9-kb *Eco*RI fragment of the wheat 45S rDNA (Gerlach and Bedbrook 1979) was directly labeled with Atto-488-11-dUTP using a nick translation kit (Jena Bioscience, Jena, Germany) and was used as a positive control probe.

### Preparation of meiotic chromosomes and FISH

Slides were prepared from plants harboring different numbers of Bs. For this, anthers of different developmental stages were fixed in 3:1 ethanol:glacial acetic acid (v/v) for 48 h at room temperature, then transferred into 70% ethanol and stored at −20 °C until processing. For the preparation of meiotic slides, fixed anthers ranging from ~0.2–0.5 mm were used. Meiocytes were squeezed out of the fixed anthers on a slide in 7 μl 1% (w/v) acetocarmine (in 45% acetic acid) with the help of a needle and squashed between the slide and coverslip as described in Windham et al. (2020). After microscopic inspection, appropriate slides were frozen in liquid nitrogen, and coverslips were removed. Slides were stored in 70% ethanol at −20 °C. FISH was applied using Fp-Sat253 and Fp-Sat2 satellite probes as described in (Ebrahimzadegan et al. 2019). For each slide, 20 μl of hybridization mixture, including 2× SSC, 50% (v/v) formamide, 20% (w/v) dextran sulfate, and 20 ng of each probe, were used. Slides were denatured on a hot plate at 80 °C for 2 min. For hybridization, slides were incubated in a humidified plastic container at 37 °C. Coverslips were removed, and slides were washed in 2× SSC for 20 min at 56 °C. Finally, slides were dehydrated and dried at room temperature, and 10 μl of Vectashield mounting medium (Vector Laboratories) containing 1 μg/ml DAPI (4′, 6-diamidino-2-phenylindole) was added to each slide as a counterstain, and a glass coverslip was applied.

### FISH using intact pollen grains

The pollen-FISH protocol described by Han et al. (2007) and Rusche et al. (1997) was optimized for *F*. *pratensis*. Briefly, yellowish mature anthers with a length of around 1 mm were fixed in 1 ml 90% acetic acid in a 1.5-ml tube for 30 min at RT. The sample was vortexed to release the pollen, and only the liquid containing pollen was transferred to a new 1.5-ml tube. Then after centrifugation at 9,000*g* for 1 min, the supernatant was removed, and the pollen pellet was kept in 70% ethanol at −20 °C. Pollen was rinsed with 1 ml 10 mM HCl two times at RT via vortexing and centrifugation (9,000*g*, 1 min). An 80 μl pepsin solution (20 mg/ml, dissolved in 10 mM HCl) was added to the pollen pellet and incubated for 30 min at 37 °C. Pollen was rinsed with 1 ml 2× SSC twice by vortexing and centrifugation (9,500*g*, 1 min), 2× SSC was removed, and 100 μl NaOH (6 mg/ml, dissolved in 70% ethanol) was added to the tube and incubated for 5 min at RT to denature the samples. After, the pollen was rinsed with 1 ml 2× SSC 3 times by vortexing and centrifugation (9,500*g*, 1 min). A 20 μl of hybridization mixture (containing 2× SSC, 50% (v/v) formamide, 20% (w/v) dextran sulfate, and 20 ng of each FISH probe) was denatured at 99 °C for 10 min and added to the prepared pollen pellet and incubated at 37 °C for 20–24 h. Pollen was rinsed in 1 ml 2× SSC by vortexing and centrifugation (9,500*g*, 1 min), and the pellet was resuspended in 1 ml 2× SSC and incubated for 30 min at 44 °C. 2× SSC was removed by centrifugation (9,500*g*, 1 min), and 15 μl DAPI (1 μg/ml in antifade solution) was added to the pellet, and 7 μl of the counterstained pollen suspension was dropped on each slide. After applying coverslips, the slides were kept overnight at 4 °C before analysis.

### Indirect immunostaining 

Root tips pretreated using nitrous oxide at a pressure of 10 bar for 2 h were fixed in 4% (w/v) paraformaldehyde (PFA) prepared in 1× phosphate-buffered saline (PBS) for 20 min on ice. Next, roots were weakly vacuumed and digested in an enzyme solution (1% pectolyase (w/v) (Sigma), 1% (w/v) cytohelicase (Sigma), 0.7% (w/v) cellulase R-10 (Duchefa), and 0.7% (w/v) cellulase (Calbiochem)) in 1× PBS for 2 h. The roots were then washed in 1× PBS on ice for 5 min twice and squashed on slides in 1× PBS + 0.001% (v/v) Tween 20 using a coverslip. Cover slips were removed after freezing in liquid nitrogen, and slides were immediately stored in 1× PBS. Immunolabeling was performed according to Manzanero et al. (2000), except that the slides were treated using a microwave 800 W for 1 min in 10 mM sodium citrate buffer to improve the chromatin accessibility (Ruban et al. 2020) and finally kept in 1× PBS for 5 min. Rabbit anti-grass CENH3 (Sanei et al. 2011) (diluted 1:2000) and donkey anti-rabbit Alexa Fluor 488 (Jackson ImmunoResearch) (diluted 1:100) were applied as primary and secondary antibodies, respectively. To distinguish between A- and B-located CENH3 signals, after immunostaining FISH using the B-specific probe, Fp-Sat253 was performed as described by Ishii et al. (2015).

### Standard fluorescence, super-resolution microscopy and measuring the size of CENH3 signals

For standard microscopy, slides were analyzed using a BX61 microscope equipped with a DP72 CCD camera (Olympus, Japan). Images were captured in black and white, pseudo-colored separately, and merged into multilayer RGB images using Adobe Photoshop (Adobe Systems, San Jose, California). To achieve super-resolution, spatial structured illumination microscopy (3D-SIM) was performed with an Elyra PS.1 microscope system equipped with a 63×/1.4 Oil Plan-Apochromat objective using the ZENBlack software (Carl Zeiss GmbH). Image stacks were captured separately for each fluorochrome using 561-, 488-, and 405-nm laser lines for excitation and appropriate emission filters (Weisshart et al. 2016). The CENH3 immuno-signal volumes, nuclear volumes, and the signal intensity per individual CENH3 volume were generated and measured with the Imaris 9.7 (Bitplane) software tool “Surface” (Randall et al. 2022).

### B chromosome size determination

To estimate the size of the B chromosome, we first measured the nuclear genome size of 0B plants of *F*. *pratensis* using flow cytometry and compared them with the genome size obtained for plants possessing 1B, 2B, or 4B chromosomes. Therefore, nuclei were isolated by chopping ~0.5 cm^2^ of young leaf tissue together with an equivalent amount of leaf tissue from the internal reference standard *Hordeum vulgare* L. convar. *vulgare *var.* hybernum* Viborg, cultivar “Hohenfinower” (Genebank Gatersleben accession number: HOR 82; 10.36 pg/2C) with a sharp razor blade in a petri dish using the CyStain PI Absolute P reagent kit (Sysmex-Partec) according to the manufacturer’s instructions. The resulting nuclei suspensions were filtered using 50-μm filters (CellTrics, Sysmex-Partec) and measured on a CyFlow Space flow cytometer (Sysmex-Partec) equipped with a 532-nm laser. Each plant was measured in total six times on 3 different days. The absolute DNA content (pg/2C) was calculated based on the values of the G1 peak means of sample and reference and the corresponding genome size (Mbp/1C) according to Dolezel (2003). Finally, the size of the B of *F*. *pratensis* was estimated by subtracting the genome size of 0B plants from the genome size of the B-possessing plants considering the number of Bs per individual.

### Flow cytometric estimation of B chromosome accumulation in sperm nuclei

Anthers with mature pollen grains were collected, and pollen nuclei were isolated in nuclei isolation buffer (Galbraith et al. 1983) applying the bursting method of Kron and Husband (2012) using CellTrics disposable filters of 100 and 20 μm (Sysmex-Partec). The propidium iodide–stained samples (PI, 50 μg/ml) were analyzed on a BD Influx cell sorter (BD Biosciences) equipped with a 488-nm and a 532-nm laser by blotting the PI fluorescence intensity (610/20) against the forward scatter (FSC) signal (488). Following the procedure of Wu et al. (2019), differences in the fluorescence intensities and FSC allowed the differentiation of vegetative and sperm nuclei without and with Bs. Based on differences in size, compaction status and RNA content vegetative nuclei show in general a higher FSC signal and an increased fluorescence intensity after staining with propidium iodide (Schoft et al. 2015).

## Results

### Meiotic B chromosome configurations at metaphase I

**Fig. 1:**
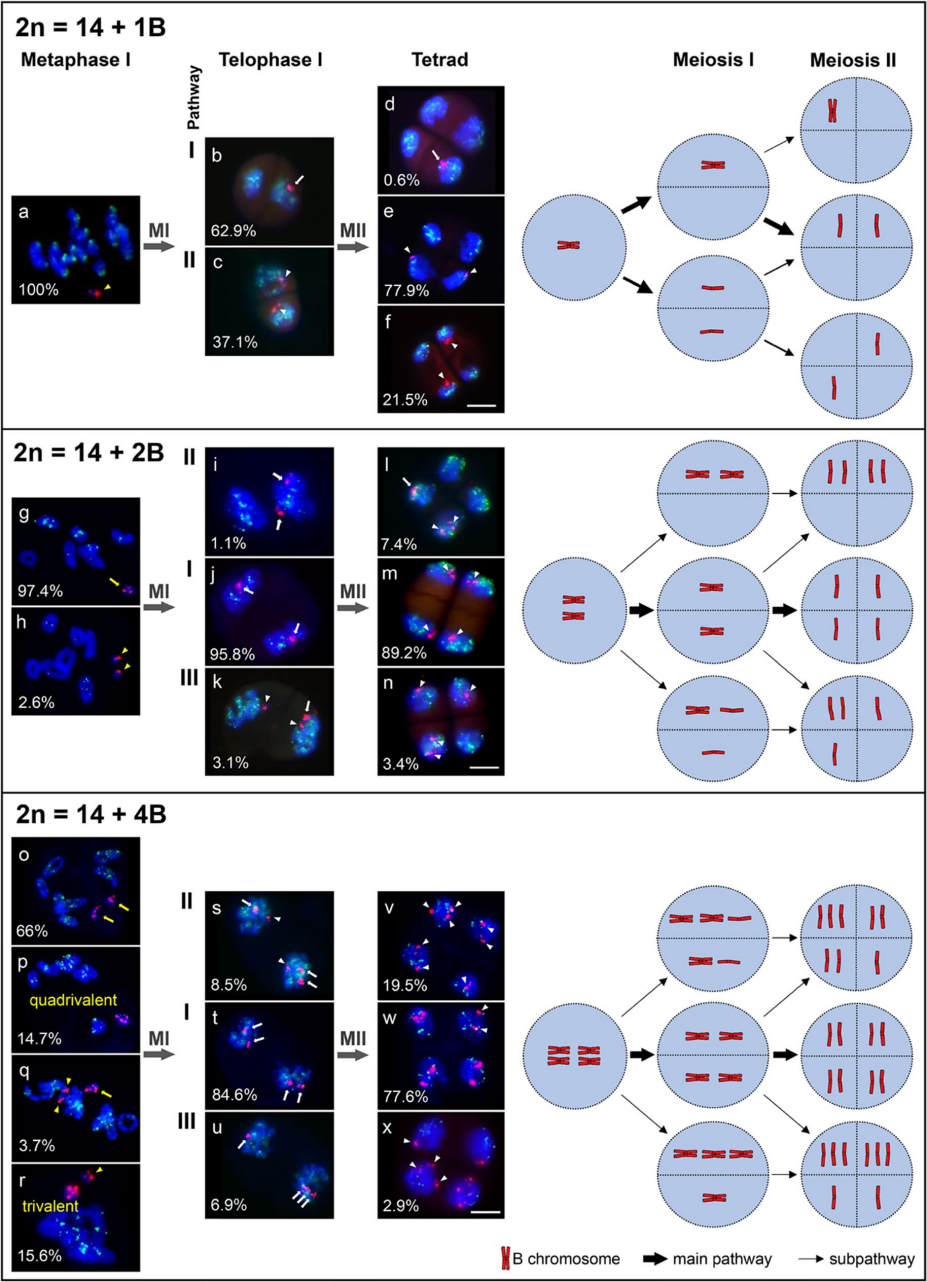
Meiotic B chromosome segregation in *F. pratensis* plants possessing different numbers of Bs (1, 2, and 4 B). 2*n* = 14 + 1B: (**a**) Metaphase I of a 1B plant showing a B univalent, (**b**) random transmission of a B chromosome to one of the poles, (**c**) separation of B chromatids during meiosis I. (**d,e**) possible B arrangements in tetrads derived from ‘b’. (**e,f**) B chromatids in tetrads resulted from ‘**c**’. Arrow in ‘**d**’ shows nondisjunction of the B chromosome in both meiosis I and meiosis II. 2*n* = 14 + 2B: Metaphase I of a 2B plant showing either one bivalent (**g**) or two B univalent (**h**). (**i**) Nondisjunction of homologous Bs and random transmission of them to one of the poles. (**j**) Normal segregation of Bs during meiosis I. (**k**) Premature separation of chromatids in one of the B chromosomes. (**l-n**) Resulting tetrads and rearrangement of B chromatids inside their microspores. 2*n* = 14 + 4B: (**o-r**) Metaphase I pairing configurations of B chromosomes of a 4B plant showing two bivalents, one quadrivalent, one B bivalents + two univalents, or one univalent + one trivalent, accordingly. (**s,u**) Irregular segregation patterns of Bs during meiosis I. (**t**) Regular segregation pattern of B bivalents during meiosis I. (**v-x**) Resultant tetrads with different arrangements of B chromatids in their microspores as a consequence of regular and irregular segregation patterns during meiosis II. The values within the images indicate the observed frequencies of each B chromosome segregation pattern. The B-specific repeat Fp-Sat253 (red) marks the B chromosome and A chromosomes were labeled with the A-specific repeat Fp-Sat2 (green). Roman numerals I, II, and III indicate the different pathways of Bs segregation during meiosis I and II. The main- and subpathways for each B segregation event are shown in the diagram corresponding to FISH images with arrows. The width of the arrows indicates the probability of the corresponding pathway. MI and MII are acronyms for meiosis I and II. Yellow arrows and arrowheads indicate B-bivalents and B-univalents, respectively. White arrows and arrowheads indicate B chromosomes and B chromatids, respectively. Scale bars = 5 μm

To track the behavior of the *F*. *pratensis* B chromosome throughout pollen formation, B-containing plants were initially identified by chromosome counting or FISH with the B-specific repeat Fp-Sat253 (Supplementary Fig. 1). Depending on the number of Bs, various B chromosome pairing configurations were observed at metaphase I ranging from univalents to quadrivalents (Fig. 1, Table 1). However, pairing between A and B chromosomes occurred in none of the cases. Briefly, in 1B plants, all Bs formed univalents (Fig. 1a). In total, 97.4% of pollen mother cells of 2B plants showed one B bivalent and rarely (2.6%) unpaired Bs (Fig. 1g, h). In 4B plants, 66% of cells revealed two B bivalents, and 14.7% possessed B quadrivalents. In addition, cells with a combination of B uni-, bi-, and/or trivalents (19.3%) were found (Fig. 1o–r). Unpaired As were never observed.

**Table 1:** Frequency of metaphase I configurations of Bs in individuals of *F*. *pratensis* with varying numbers of B chromosomes

Plants	Univalent	Bivalent	Two univalents + two bivalents	One univalent + one trivalent	Quadrivalent
1B (100)*	100%	0	0	0	0
2B (78)	2.6%	97.4%	0	0	0
4B (109)	0	66%	3.7%	15.6%	14.7%

*Numbers inside the parenthesis indicate the number of cells analyzed

### Meiotic B chromosome segregation in 1B plants

To study the meiotic segregation of single Bs, four 1B plants (three plants from the greenhouse and one from the field) and for each plant, more than three spikes were examined (Fig. 1, Supplementary Table 1). During meiosis I, B univalents showed two different segregation pathways. In total, 62.9% of the B univalents were transmitted to one of the poles (pathway I, Fig. 1b), and 37.1% of the B univalents prematurely separated into sister chromatids (pathway II), hence both telophase I cells obtained B chromatids (Fig. 1c).

In meiosis II, both B chromatids of a univalent may or may not separate; consequently, the resulting tetrads exhibited three different B configurations. In the most frequently found configuration 1 (77.9%), two B chromatids locate in adjacent microspores (Fig. 1e), which can result from both pathways of B segregation in meiosis I (Fig. 1b, c). In configuration 2, two B chromatids locate in alternate microspores (Fig. 1f), as a result of pathway II (Fig. 1c). In the rarely occurring configuration 3 (0.6%), likely due to nondisjunction of B chromatids during meiosis II, the inclusion of both chromatids into one of the microspores occurred (Fig. 1d).

### Meiotic B chromosome segregation in 2B plants

To study the meiotic segregation of a pair of Bs, three 2B plants (two plants from the greenhouse and one from the field) and for each plant, more than three spikes were examined (Fig. 1, Supplementary Table 1). The Bs of 2B plants migrated in three different segregation pathways during meiosis I. In pathway I, both B homologs prevalently segregated normally to opposite poles with a frequency of 95.8% (Fig. 1j), and the majority (89.2%) of the resulting tetrads contained one B chromatid in all four microspores (Fig. 1m, Supplementary Fig. 2b, d). In addition, this pathway could also result in configurations in which the tetrads have either 0 or 2B chromatids (Fig. 1l), or 0, 1, or 2B chromatids (Fig. 1n) in cases when the chromatids of the Bs of both or one B chromosome, respectively, are not segregating in meiosis II. In pathway II, B bivalents did not segregate; in some cases, they lagged in anaphase I (Supplementary Fig. 2c) and were consequently included in one of the two daughter cells at the end of meiosis I (Fig. 1i). This pathway happened with a frequency of 1.1% and results in the depletion of Bs in half of the microspores, while the other half includes 2B chromatids at the end of meiosis II (Fig. 1l, Supplementary Fig. 2e). In pathway III, in 3.1% of cells, the chromatids of one of the Bs prematurely separated, while the chromatids of the second B remained cohered. As a result, one daughter cell received three and the other one B chromatid at the end of meiosis I (Fig. 1k). Consequently, microspores of the resulting tetrads received 0, 1, or 2B chromatids (Fig. 1n).

### Meiotic B chromosome segregation in a 4B plant

Three different pathways of meiotic B segregation were observed in a 4B plant. In most cases (pathway I), the B chromosomes followed the standard segregation in meiosis I (84.6%) and meiosis II (77.6%), resulting in the inclusion of most likely two non-sister B chromatids into each microspore of a tetrad (Fig. 1t, w). In pathway II of meiosis I, which occurred with a frequency of 8.5%, two Bs with nonseparated sister chromatids plus one single-chromatid B pulled to one pole, and the remaining Bs (one B with nonseparated sister chromatids and a B chromatid) migrated to the opposite poles in telophase I (Fig. 1s). This resulted in microspores containing 1, 2, or 3 B chromatids (Fig. 1v). Since the percentage of such tetrads is higher (19.5%) than that of pathway II (8.5%), some of them seem to be the result of pathway I. In total, 6.9% of cells showed pathway III. This abnormal segregation of Bs during meiosis I led to one cell with 3 Bs and the opposite one with 1 B (Fig. 1u). As a result, tetrads possessing two microspores with 3 Bs and the other ones only 1 B chromatid were observed in 2.9% of the analyzed tetrads (Fig. 1x). In some cases (e.g. Fig. 1u compared to 1x), the detected frequency of the meiosis II configuration deviated from what should be expected according to the observed frequency in meiosis I. This most likely arises from the fact that meiosis I and II were independently evaluated using different anthers and/or sample sizes.

### Lagging chromosomes and micronuclei

To evaluate the occurrence of lagging chromosomes and/or micronuclei, more than 180 anaphase I cells and tetrads of each B-containing individual were analyzed (Supplementary Table 2). Although we observed laggards of both A and B chromosomes, the number of lagging Bs at anaphase I was at least four times higher than that of lagging As (Supplementary Fig. 3, Supplementary Table 2). Depending on the number of Bs in the individual, we found different combinations of B chromatids and chromosome laggards (Supplementary Fig. 3a-l) with 4B pollen meiocytes showing the highest total number of laggards (Supplementary Table 2). However, at the end of meiosis, only a low number of tetrads containing micronuclei, either with or without Bs, were observed (Supplementary Fig. 4, Supplementary Table 2). Thus, lagging Bs formed micronuclei less often than expected.

### The mechanism of B chromosome accumulation during pollen mitosis

**Fig. 2:**
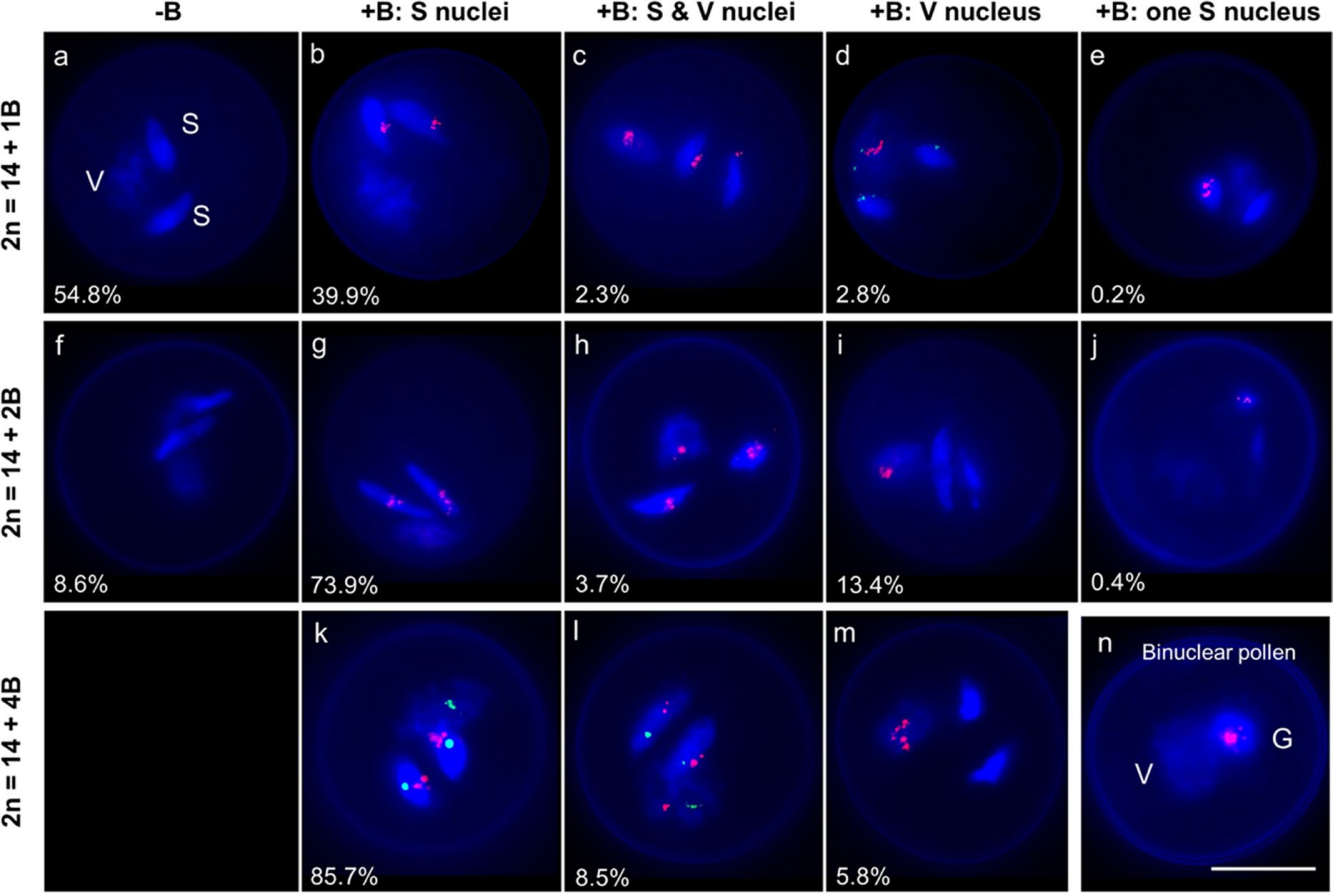
Analysis of mature pollen grains of *F*. *pratensis* plants possessing 1, 2, or 4 B chromosomes by fluorescence in situ hybridization (FISH) with the B-specific repeat Fp-Sat253 in red (**a–c**, **e**, **f–j**, **m**, **n**), the combination of Fp-Sat253 (red) and A-specific repeat Fp-Sat2 in green (**d**), and in the combination of Fp-Sat253 (red) and 45S rDNA in green as a control (**k**, **l**). Binuclear pollen in (**n**) shows the drive of the B chromosome toward the generative nucleus after the first pollen mitosis. The shown values indicate the frequency of the observed B accumulation patterns. V, S, and G are acronyms for vegetative, sperm, and generative nuclei, respectively. Scale bar = 5 μm

The post-meiotic behavior of *F*. *pratensis* Bs in mature pollen grains of 1B, 2B, and 4B plants was analyzed by FISH using the B-specific probe Fp-Sat253 and quantitative flow cytometry. The A-specific probes Fp-Sat2 or 45S rDNA were used as a FISH-positive control, and at least 500 pollen grains per genotype were analyzed by FISH (Table 2). The contrasting morphology of vegetative and sperm nuclei allowed the identification of both nuclei types. Vegetative nuclei were decondensed and round, while sperms were condensed and spindle-shaped (Fig. 2a).

**Table 2:** Frequency of *F*. *pratensis* Bs accumulation in sperm nuclei and drive of Bs

Plants	*Drive (%) (Number of nuclei counted)	**Frequency of Bs in sperm nuclei (%)		
		0B	2B	4B
1B	88.7 (712)	69.6	30.4	0
2B	82 (519)	17.1	82.9	0
4B	85.7 (601)	8.1	33.6	58.3

*Frequency of drive was determined by FISH

**Frequency of B chromosome (Bs) accumulation in sperm nuclei was determined by flow cytometry

In 1B plants, 54.8% of mature pollen were B-negative (Fig. 2a), and 39.9% showed B-specific signals in both sperm nuclei (Fig. 2b). In total, 2.3% of pollen showed B-specific signals in all three nuclei (Fig. 2c), 2.8% in vegetative nuclei only (Fig. 2d), and 0.2% in only one of both sperms (Fig. 2e).

In 2B plants, 8.6% of pollen grains lacked Bs (Fig. 2f). In total, 73.9% of B-positive pollen contained Bs in only both sperms (Fig. 2g), and 3.7% showed signals in all three nuclei (Fig. 2h). In 13.4% Bs were only observed in the vegetative nucleus (Fig. 2i), and 0.4% Bs located only in one of the sperm nuclei (Fig. 2j).

In the 4B plant, all examined pollen grains were B positive, and three different B distributions were observed. Most pollen grains (85.7%) revealed B signals only in both sperms (Fig. 2k). In total, 8.5% showed Bs in all three nuclei (Fig. 2l), and 5.8% showed Bs only in the vegetative nucleus (Fig. 2m).

Based on the observed segregation patterns of Bs, it is evident that nondisjunction of Bs occurs at first pollen mitosis resulting in a preferential accumulation of Bs in the generative nucleus (Fig. 2n). The drive frequency was quantified by dividing the number of pollen in which Bs preferentially accumulated in sperm cells by the total number of B-positive pollen (Tables 2 and 3). In total, Bs accumulated in sperm nuclei with a frequency of at least 82%.

**Table 3:** Comparison of FISH and flow cytometry (FC) methods for estimating B chromosome (Bs) accumulation in sperm nuclei

Plants	Methods	Sperms without Bs (%)	Sperms with Bs (%)
1B	FISH / FC	57.8 / 69.6	42.2 / 30.4
2B	FISH / FC	22.2 / 17.1	77.8 / 82.9
4B	FISH / FC	5.8 / 8.1	94.2 / 91.9

### Quantification of B chromosome accumulation in sperm nuclei by flow cytometry

**Fig. 3:**
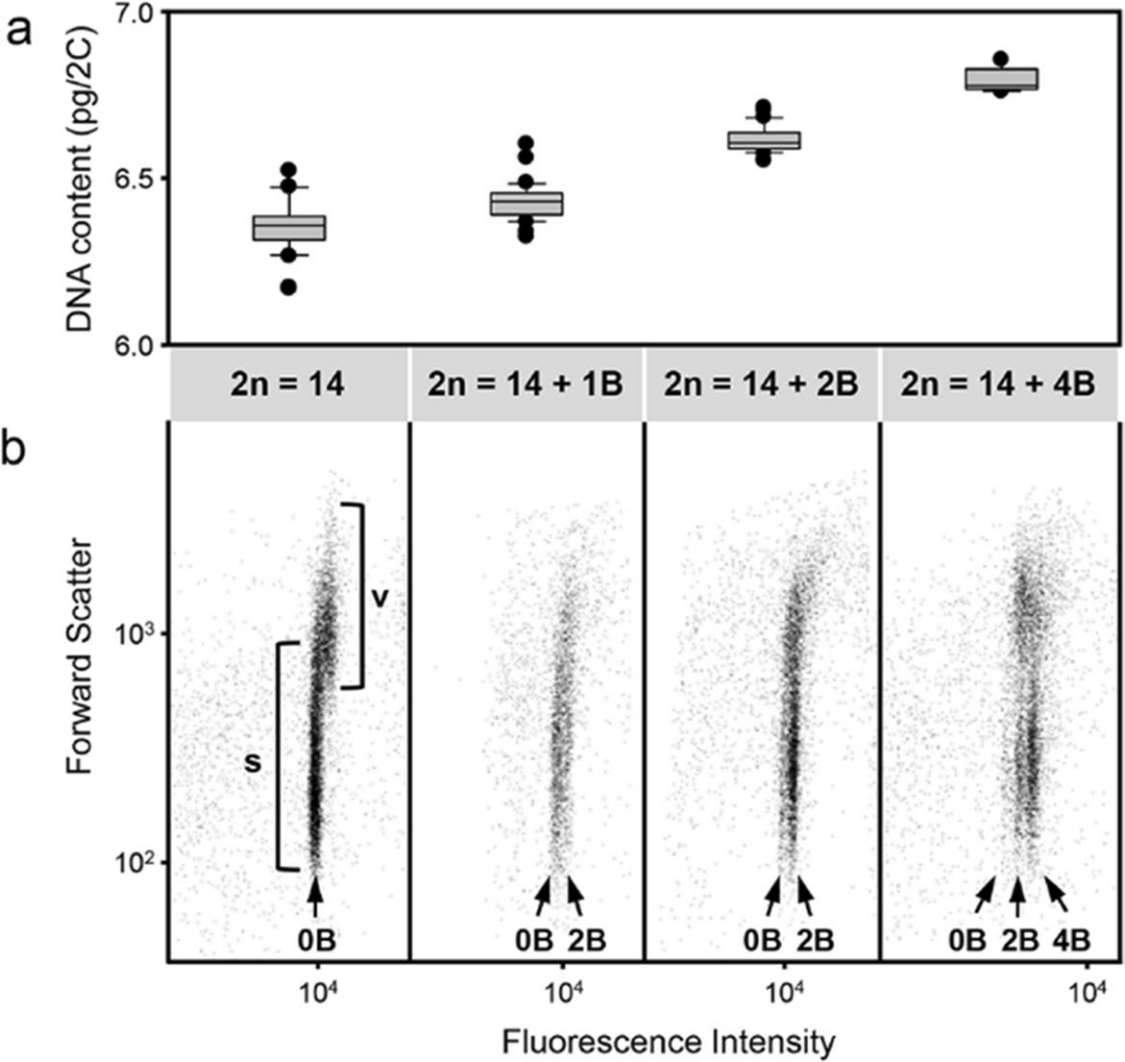
Flow cytometric analysis.** a** Measurements of the DNA content of plants with different numbers of B chromosomes to estimate the size of the *F*. *pratensis* B chromosome (103 Mbp). **b** Flow cytometric estimation of B chromosome distribution in sperm nuclei in individuals of *F*. *pratensis* possessing 1, 2, and 4 Bs. Dot plot showing the relative fluorescence (DNA content; *x*-axis) vs forward scatter signal (particle size; *y*-axis) intensities. Flow cytometry showed one additional nuclei population in the case of 1B and 2B individuals and two additional populations in the case of a 4B plant. All nuclei populations are indicated by arrows. Note: Due to differences in size, compaction status, and RNA amount (Schoft et al. 2015) the vegetative nuclei are characterized by increased forward scatter and fluorescence intensity signals. This is especially obvious in the 0B plant while in B-containing plants, the difference in the fluorescence intensity between nuclei types is compensated by an increased DNA content in the sperm nuclei. V and S are acronyms for vegetative and sperm nuclei, respectively

Flow cytometry allows differentiating between vegetative and sperm nuclei as well as B-positive and B-negative nuclei isolated from mature pollen grains according to differences in the fluorescence intensity and forward scatter (FSC) signals, representing the DNA content and the size of the nuclei, respectively. Furthermore, it enables the precise determination of the B chromosome number in the corresponding nuclei types in high throughput. The number of Bs in each population can be estimated by the distance between the 0B nuclei population and corresponding +B populations (Fig. 3b), based on the calculated size of the B chromosome in *F. pratensis* of around 103 Mbp (Fig. 3a). Flow cytometric analysis of isolated nuclei from pollen grains of mother plants with 1, 2, or 4 Bs showed in addition to the populations of vegetative and sperm nuclei observed in a 0B plant further populations of B-containing sperm nuclei with a higher fluorescence intensity relative to the 0B sperm nuclei (Fig. 3b).

Pollen nuclei from 1B mother plants showed an additional fraction of sperm nuclei at a location corresponding to two B chromosomes; however, 69.6% of sperms lacked Bs, and the rest (30.4%) contained 2 Bs (Fig. 3b, Table 2). This result agrees with the segregation patterns of 1B individuals during meiosis where the B appeared as univalent in metaphase I and its segregation patterns during meiosis I (Fig. 1a-c), finally resulting in tetrads in which only two out of the four spores contain 1 B each (Fig. 1e, f).

Flow cytometry of pollen nuclei of 2B mother plants revealed 82.9% of sperm nuclei to contain two Bs (Table 2, 2B mother plants) and the rest (17.1%) 0 B. In PMCs of 2B plants, the 2 Bs mainly formed a bivalent in metaphase I (FISH analysis; Fig. 1g) and segregated normally during meiosis, producing spores each containing one B (Fig. 1m). The subsequent mitotic divisions in these spores finally lead to pollen grains with sperm nuclei having preferentially 2 Bs as a result of the drive mechanism.

Pollen nuclei of the 4B plant resulted in two additional fractions of sperm nuclei (besides the 0B fraction) corresponding to nuclei with either two or four additional B chromosomes (Fig. 3b). Here, the majority of sperm cells (58.3%) contained four Bs, 33.6% contained two Bs, and 8.1% lacked B chromosomes (Table 2, 4B mother plant). Such an accumulation of Bs in sperm cells could result from various patterns of B chromosome pairing during metaphase I resulting in univalent to quadrivalent configurations (Fig. 1o–r) that collectively formed different combinations of spores with three, two, and one B chromatids (Fig. 1v–x). As expected, most spores contained two Bs that finally contributed to the formation of sperm nuclei, each containing four Bs. However, due to the small size of the B chromosome in *F*. *pratensis*, we cannot exclude that we failed to detect nuclei with deviating B chromosome numbers occurring in low proportions. Our results are in agreement with Mendelson and Zohary (1972) who reported the most frequent occurrence of normal segregation of B chromosomes for 4B plants during meiosis.

### B chromosomes contain less centromeric CENH3 than A chromosomes

**Fig. 4:**
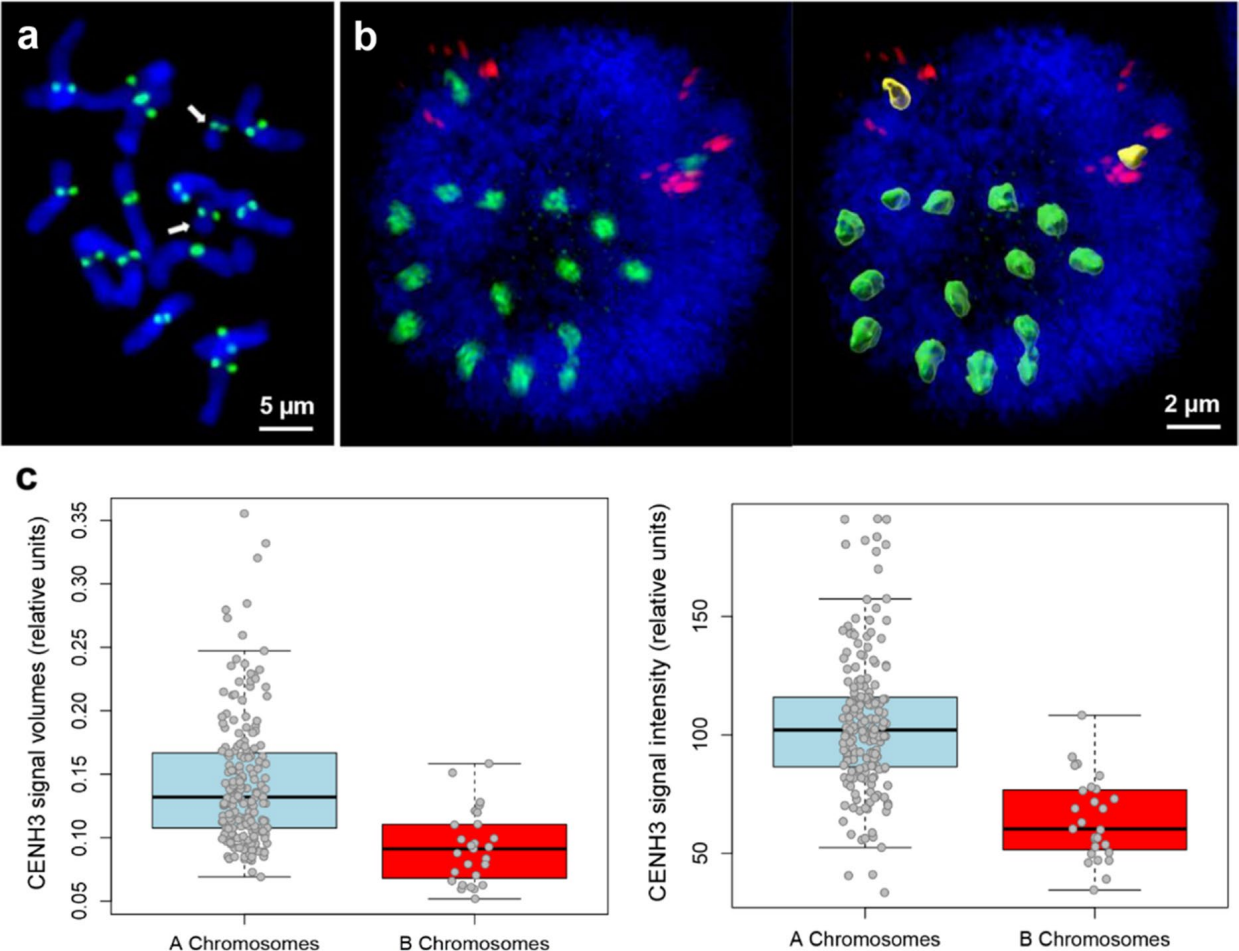
CENH3 immuno-signal evaluation on metaphase chromosomes and root nuclei of *F*. *pratensis* containing 2Bs. **a** All chromosomes show CENH3 double signals at their A and B (arrows) centromeres. **b** CENH3 signal volume measurements on an interphase nucleus after surface rendering (right). The B chromosomes are marked by FISH using the B-specific repeat Fp-Sat253 (red). Most CENH3 volumes and the signal intensities inside of the Bs (yellow) are smaller than those of the As (green) (Supplementary Table 3). **c** Relative CENH3 signal volumes (left) and intensities (right) for As and Bs of 14 root nuclei. The box contains the data between the upper and lower quartile, the line within the box marks the median. The whiskers above and below the box indicate the 90th and 10t percentiles. To compensate for differences between the individual nuclei, the signal volumes were normalized with respect to the nuclear volumes (signal volume (normalized) = signal volume (raw)/nuclear volume (raw) * 100) and the intensities according to the following formula: intensity (normalized) = intensity (raw)/average intensity per nucleus * 100

Because centromere activity and the relative CENH3 quantity are associated (Howman et al. 2000; Marimuthu et al. 2021; Sanei et al. 2011), we determined whether A and B chromosomes contain similar or different CENH3 amounts. To distinguish between A and B centromeres, we labeled root nuclei with FISH using the B-specific repeat Fp-Sat253 and also applied anti-CENH3 antibodies. 3D-SIM image stacks were used to perform surface rendering of the CENH3 signals and the DAPI-labeled nuclei. Most of the Bs showed smaller CENH3 volumes and lower signal intensities inside compared to As (Fig. 4b, c, Supplementary Table 3). Statistical analysis confirmed that both the relative CENH3 signal volumes and intensities are significantly different (*P* < 0.001; non-parametric Mann-Whitney *U* test) between Bs and As. This demonstrates that the B centromeres are smaller and contain less CENH3 than those of As. However, whether Bs of pollen nuclei show a similar reduction in the amount of CENH3 and whether this is involved in B chromosome drive remains to be elucidated.

## Discussion

FISH analysis of trinuclear pollen grains revealed that the drive of Bs mainly occurs during the first pollen mitosis in *F. pratensis*, when Bs preferentially integrate into the generative nucleus. During the second pollen mitosis, most Bs segregate equally to sperm nuclei. However, in 1B and 2B plants, there were a few occurrences (< 0.5%) of uneven B segregation resulting in pollen grains with B-specific signals in only one of the two sperm nuclei.

We did not find 6B-containing sperm cells as theoretically expected from microspores of a 4B plant with three Bs (Fig. 1v, x). It seems that the accumulation of more than 4 Bs inside sperms rarely happens in the plant we studied. Also, 1B plants showed a slightly higher drive efficiency than 2B and 4B individuals (89% vs 82% and 85%). Thus, a maximum number of Bs per sperm may exist. The upper limit is species-specific, as different species can tolerate various ranges of Bs. For instance, maize and rye plants can accept up to 34 and 6 Bs, respectively (Jones 1995). In *F. pratensis*, individuals with up to five Bs have been reported (Bosemark 1950; Bosemark 1954). The B chromosome number capacity may be generally determined by a balance between the efficiency of drive and the detrimental effect of Bs on fertility and development, which may be dosage dependent.

The distantly related species rye and *A*. *speltoides* possess a similar process of B drive as observed in *F*. *pratensis*. Also, the frequency of B chromosome accumulation in generative nuclei is almost comparable. In *Ae*. *speltoides* (Wu et al. 2019) and rye (Niwa and Sakamoto 1995, 1996), Bs accumulate in more than 93% of generative nuclei, while in *F*. *pratensis*, Bs enriched in up to 89% of generative nuclei. The accumulation of Bs in generative nuclei of rye and *Ae*. *speltodies* is caused by a combination of B chromosomal nondisjunction and asymmetrical spindle structure (Banaei-Moghaddam et al. 2012; Endo et al. 2008; Wu et al. 2019). Interestingly, the Bs of all these species are enriched in B-specific satellite DNA (Ebrahimzadegan et al. 2019; Klemme et al. 2013; Wu et al. 2019). It has to be elucidated whether these repeats, as hypothesized by Camacho et al. (2021), are components of the drive mechanism, as previously documented for the segregation distortion system in *D*. *melanogaster* (Larracuente 2014).

The *F*. *pratensis* B centromeres revealed a reduced amount of CENH3 in root nuclei. Since the position of centromeres is epigenetically determined by CENH3, alteration of CENH3 quantity in some organisms has been accompanied by chromosome nondisjunction and various chromosome segregation errors (Buchwitz et al. 1999; Lermontova et al. 2011; Stoler et al. 1995). However, despite a reduced CENH3 amount in the B centromeres, we did not observe disturbed mitotic divisions or micronuclei in root meristems of *F*. *pratensis*. Whether a similar difference in the CENH3 amount between As and Bs exists in pollen undergoing the first mitosis is unknown. On the other hand, no differences in the amount of CENH3 were found between the As and Bs of rye and *Ae*. *speltoides* (Banaei-Moghaddam et al. 2012; Wu et al. 2019). In both species, B-specific nondisjunction occurs during the first pollen mitosis, suggesting that differences in the CENH3 quantity are not necessarily the reason for nondisjunction during the first pollen mitosis, and other mechanisms must be involved in this process.

Comparing the results of flow cytometry and pollen FISH revealed that the estimated accumulation frequencies by both methods were in close proximity. For example, in the evaluated 2B plants, the frequency of B accumulation in sperm nuclei detected by FISH was 77.8%, and flow cytometry resulted in only a slightly higher number of B-containing sperm nuclei (82.9%). This low difference may be due to sampling size differences used for the analysis. Each approach has its specific advantages. Flow cytometry allows the analysis of a large number of sperm nuclei for the presence of Bs and the estimation of the number of Bs per nucleus. For instance, in an analyzed 4B plant, three different fractions of sperms were identified with 0B, 2B, and 4B at different frequencies. FISH, as a supplemental method, indicates the physical location of Bs inside nuclei of individual pollen grains and may be used to determine the stage of pollen formation as well as the frequency of B drive.

Some B chromosomes use non-Mendelian ways to pass through the meiosis in *F*. *pratensis*. Thus, besides nondisjunction during pollen mitosis also, the meiotic segregation behavior of Bs can contribute to the accumulation of Bs in microspores that consequently affect the number of Bs in gametes and resulting offspring. In 1B plants, premature separation of sister chromatids or segregation of the whole univalent to one of the poles during meiosis I results mostly in tetrads without a B in half of the microspores (Fig. 1e, f). However, if also during meiosis II, nondisjunction of the B chromatids occurs, Bs are accumulated in a tiny fraction (0.6%) of tetrads (Fig. 1d).

Generally, a univalent is easy to eliminate from meiosis, and for example, in monosomic wheat genotypes, the transmission rate of A chromosomes is reduced to about 15–45% (Tsunewaki 1964). Mendelson and Zohary (1972) suggested two meiotic scenarios for *Ae*. *speltoides* plants with 1B. First, a B univalent behaves as a laggard during meiosis, which results in a micronucleus, excluding the Bs from the daughter nuclei. Second, in around 20% of cells, the B migrates to one of the poles. Surprisingly, in our analyses, almost two-thirds of the telophase I cells contained the univalent in one daughter nucleus. We also observed lagging B univalents and some micronuclei during meiosis, but FISH demonstrated that in the case of 1B plants, only A chromosomes formed micronuclei (Supplementary Fig. 4a-d).

Studies have shown that two or more homologous Bs pair in different manners, resulting in configurations ranging from bivalent to multivalent. Unpaired Bs are capable of passing through meiosis as univalent and finally might contribute to meiotic accumulation of Bs (Datta et al. 2016; Jones and Houben 2003; Jones 1991). Most Bs of *F*. *pratensis* in 2B and 4B individuals formed bivalents (Fig. 1g, o) and segregated like A chromosomes (Fig. 1j, m, t, w). Camacho (2005) and Camacho et al. (2000) proposed if Bs form bivalents, they do not accumulate or undergo loss because they segregate normally to opposite poles. We indeed found that in 2B plants, 95.8% of B bivalents performed normal segregation. Still, sometimes, specifically in plants grown under field conditions, paired Bs remained jointed and transmitted to one of the poles and consequently accumulated (Fig. 1i). These findings were consistent with those of Komluski et al. (2022), who discovered that nondisjunction of paired accessory chromosomes in fungi during the first meiotic division led to the accumulation of Bs in half of the offspring and deprivation of Bs in the remaining spores. Nonseparation of bivalents (Fig. 1s, u) also led to the accumulation or depletion of Bs in tetrads in 4B plants (Fig. 1v, x).

The accumulation of *F*. *pratensis* Bs during meiosis differs from the meiotic drive of Bs found in other species, because meiotic drive is typically determined by the functional asymmetry in meiocytes. In female meiosis, for example, asymmetry leads to the formation of just one functional gamete, and drive occurs when Bs are preferentially transferred to the functional megaspore (Jones 2018). On the male side, however, all four resultant microspores have an equal probability of producing mature pollen via successive mitotic divisions, demonstrating a lack of meiotic drive. We analyzed the frequency of microspores with varied numbers of Bs in a 4B plant to gain a better understanding of the meiotic accumulation of Bs in *F*. *pratensis*. Interestingly, both 1B- and 3B-containing microspores had an equal frequency of passing through the post-meiotic division of 6.3% (Fig. 1v, x and the corresponding diagram).

In summary, most Bs of *F*. *pratensis* segregate during meiosis like standard A chromosomes, although there are some exceptions where Bs use non-Mendelian ways to pass through the meiosis, we defined them as meiotic accumulation. However, a true drive of Bs happens during the first pollen mitosis, by which Bs preferentially migrate to the generative nucleus.

### Supplementary information

Supplementary file 1 (DOCX 2.09 MB)

## Abbreviations

As: A chromosomes
Bs: B chromosomes
CENH3: Centromere-specific histone H3
FISH: Fluorescence in situ hybridization
G: Generative nucleus
h: Hour
min: Minute
PMCs: Pollen mother cells
rpm: Rotations per minute
RT: Room temperature
S: Sperm nucleus
V: Vegetative nucleus
