# Supplementary material for: Meiotic segregation and post-meiotic drive of the Festuca pratensis B chromosome
Source: Chromosome Res. 2023 Sep 2;31(3):26. doi: 10.1007/s10577-023-09728-6 (PMC10474989; doi:10.1007/s10577-023-09728-6)
Supplement: Supplementary file 1 — (DOCX 2.09 MB) [file 10577_2023_9728_MOESM1_ESM.docx]

**Supplementary Materials**


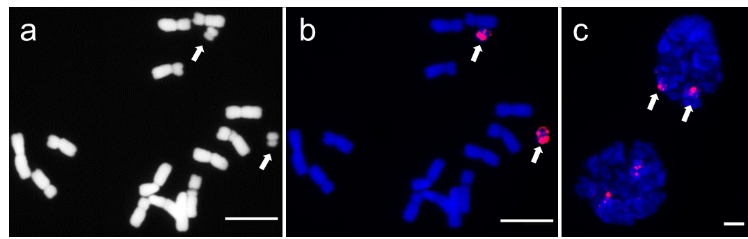


**Supplementary Fig. 1** *F. pratensis* cells with 2 B chromosomes. (**a**) Mitotic metaphase chromosomes before and (**b**) after FISH using the B-specific probe Fp-Sat253 (red). (**c**) Interphase nuclei after FISH using the B-specific probe Fp-Sat253 (red). Arrows show B chromosomes. Scale bar = 5 µm


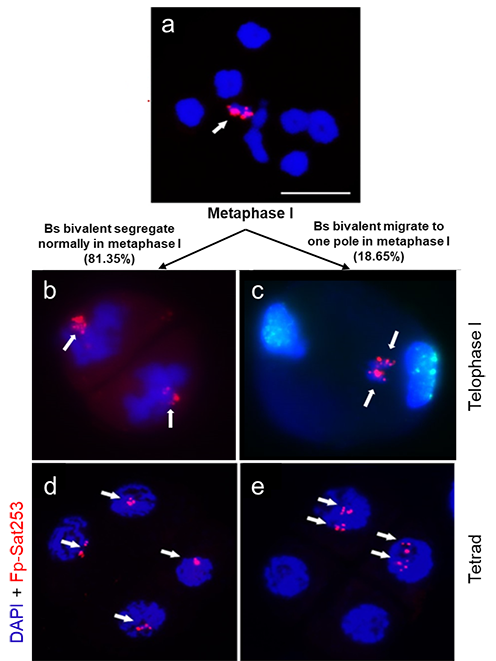


**Supplementary Fig. 2** Behavior of B chromosomes in plants grown under field conditions possessing 2 Bs. The two Bs form a bivalent at metaphase I (arrow in **a**), which in 81.35% of the meiocytes segregates to the daughter cells normally (arrow in **b**). Because the chromatids segregate correctly during meiosis II, the outcome would be four microspores each containing one B (**d**). In 18.65% of meiocytes, Bs bivalent migrates to one pole during anaphase I (arrow in **c**). Consequently, 50% of the resulting microspores in this pathway contain two Bs, while the second half was B negative (**e**). Arrows show Bs after FISH with the B-specific repeat Fp-Sat253 (red)

**
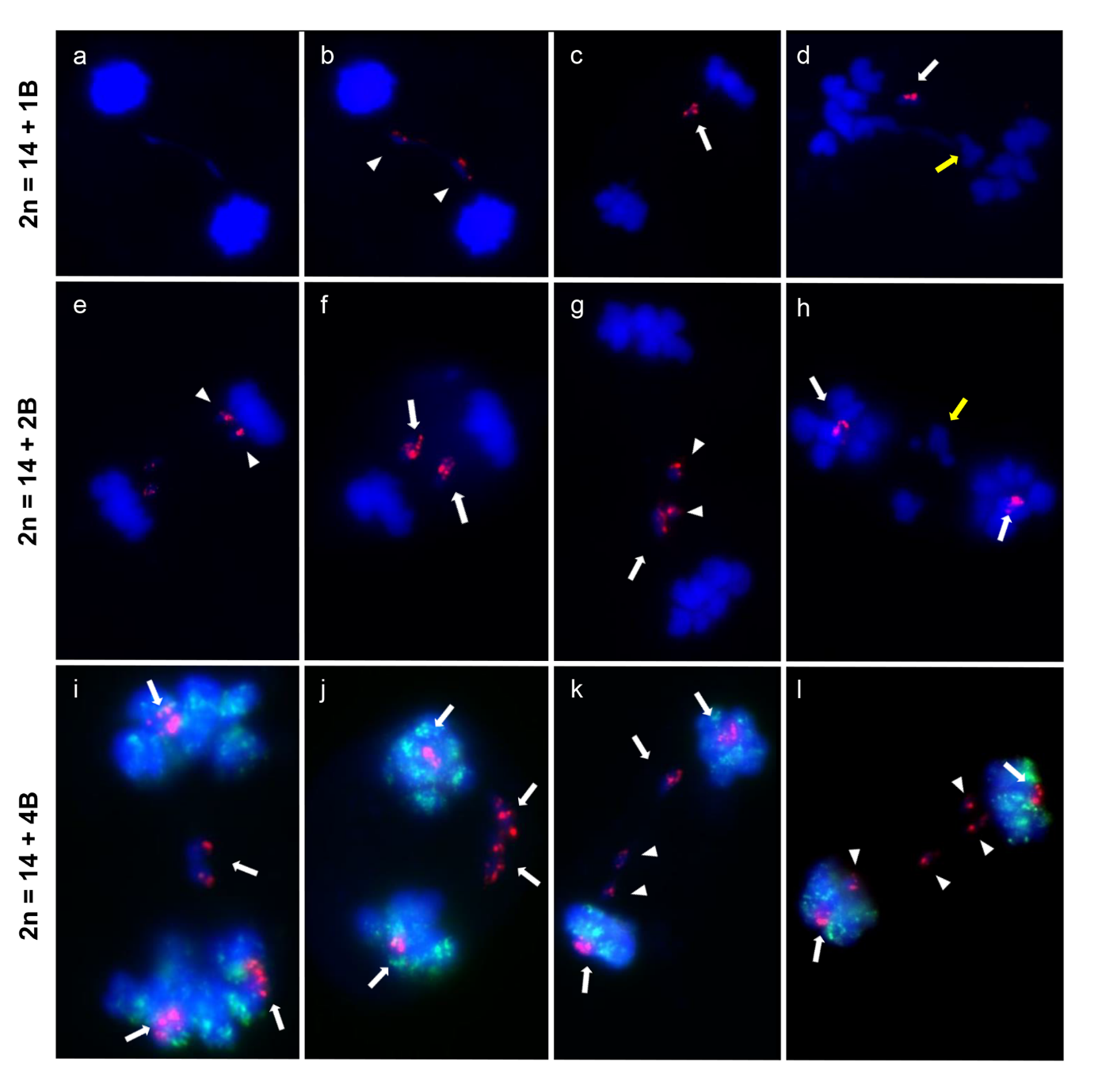
**

**Supplementary Fig. 3** Different combinations of B chromosome laggards at anaphase I of *F. pratensis.* (**a-c**) B chromatid/chromosome laggards and (**d**) a combination of A and B chromosome laggards in a 1B plant. (**e,f**) B chromatid/chromosome laggards, (**g**) a combination of B chromatid and chromosome laggards, and (**h**) A chromosome laggards in a 2B plant. (**i,j**) show one or two lagging B chromosomes, (**k,l**) show lagging Bs with one and two chromatids in a 4B plant. Fp-Sat253 (red) shows Bs and As labeled with A-specific repeat Fp-Sat2 (green). White arrows and arrowheads represent lagging Bs and B chromatids, respectively. Yellow arrows show lagging As


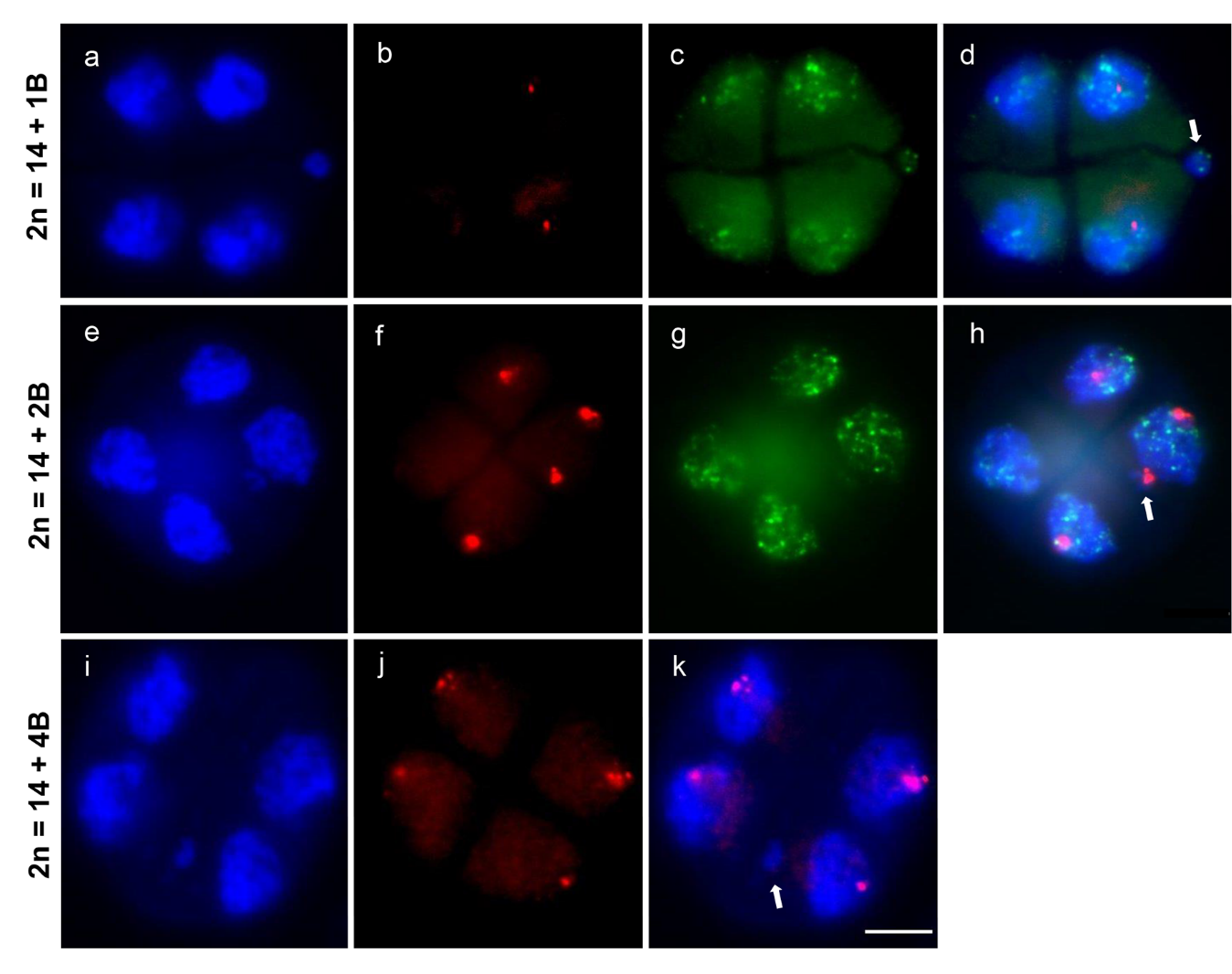


**Supplementary Fig. 4** Occurring micronuclei in *F. pratensis* plants with different numbers of B chromosomes. (**a-d** and **i-k**) B chromosome-negative micronuclei produced in 1B and 4B individuals. (**e-h**) Micronuclei found in 2B plants contain Bs

**Supplementary Table 1** Growth conditions, number of analyzed plants, and number of meiosis I cells per plant analyzed

| Growth conditions | Number of Bs | Number of plants;  Cells in meiosis I | Number of plants; Cells in meiosis II |
| --- | --- | --- | --- |
| Field | 1B | 1; 100 | 1; 100 |
|  | 2B | 1; 100 | 1; 100 |
| Controlled | 1B | 3; 742 | 2; 431 |
|  | 2B | 2; 1100 | 2; 1086 |
|  | 4B | 1; 578 | 1; 321 |

**Supplementary Table 2** Number of cells with A or B laggards in anaphase I and formed micronuclei

| Number of laggards in anaphase I | | | |
| --- | --- | --- | --- |
| Mother plants | Number of cells | Lagging A | Lagging B |
| 1B | 186 | 1.1% | 4.8% |
| 2B | 347 | 0.9% | 3.8% |
| 4B | 260 | 0.8% | 6.2% |
| Number of micronuclei in tetrads | | | |
| Mother plants | Number of cells | Micronuclei -B | Micronuclei +B |
| 1B | 186 | 2.7% | 0 |
| 2B | 347 | 0.9% | 0.6% |
| 4B | 260 | 0.8% | 0 |

**Supplementary Table 3** CENH3 signal volumes (µm³) and intensities of A and B chromosomes detected in 14 root nuclei of *F. pratensis*. The strong nucleus, CENH3 volume and signal intensity differences are due to measuring nuclei of different cell cycle stages (G1, S and G2). Values of A chromosomes are in black, those of Bs are in red. Nucleus 1 contains only 1B. All other nuclei are 2n=14+2Bs. Missing A values are due to associated signals which could not be measured separately. The intensity values represent the sum of intensities evident within the voxels of the CENH3 signal volumes. Individual CENH3 volume and intensity values are ranked according to increasing size

| **Nucleus** | **1** | **2** | **3** | **4** | **5** | **6** | **7** | **8** | **9** | **10** | **11** | **12** | **13** | **14** |
| --- | --- | --- | --- | --- | --- | --- | --- | --- | --- | --- | --- | --- | --- | --- |
| **Nucleus volume (µm³)** | 630 | 718 | 576 | 701 | 502 | 332 | 615 | 388 | 719 | 673 | 623 | 495 | 384 | 354 |
| **CENH3 volumes (µm³)** | 1.00 | 0.65 | 0.36 | 0.67 | 0.49 | 0.33 | 0.54 | 0.20 | 0.87 | 0.40 | 0.37 | 0.31 | 0.27 | 0.28 |
|  | 1.04 | 1.04 | 0.42 | 0.87 | 0.56 | 0.36 | 0.57 | 0.26 | 0.90 | 0.41 | 0.52 | 0.39 | 0.36 | 0.34 |
|  | 1.11 | 1.09 | 0.49 | 0.88 | 0.56 | 0.38 | 0.66 | 0.27 | 0.92 | 0.56 | 0.54 | 0.42 | 0.46 | 0.39 |
|  | 1.33 | 1.18 | 0.49 | 0.88 | 0.64 | 0.39 | 0.67 | 0.28 | 0.97 | 0.57 | 0.55 | 0.42 | 0.53 | 0.40 |
|  | 1.34 | 1.35 | 0.53 | 0.92 | 0.67 | 0.40 | 0.70 | 0.32 | 1.08 | 0.60 | 0.56 | 0.47 | 0.53 | 0.40 |
|  | 1.35 | 1.40 | 0.55 | 0.95 | 0.74 | 0.42 | 0.72 | 0.32 | 1.09 | 0.61 | 0.62 | 0.48 | 0.56 | 0.40 |
|  | 1.44 | 1.42 | 0.73 | 0.99 | 0.79 | 0.42 | 0.72 | 0.32 | 1.10 | 0.61 | 0.64 | 0.49 | 0.56 | 0.42 |
|  | 1.44 | 1.50 | 0.74 | 0.99 | 0.86 | 0.44 | 0.74 | 0.35 | 1.10 | 0.62 | 0.65 | 0.50 | 0.60 | 0.45 |
|  | 1.46 | 1.57 | 0.75 | 1.03 | 0.94 | 0.46 | 0.77 | 0.35 | 1.11 | 0.64 | 0.65 | 0.50 | 0.64 | 0.45 |
|  | 1.49 | 1.60 | 0.76 | 1.03 | 0.96 | 0.46 | 0.79 | 0.36 | 1.18 | 0.64 | 0.67 | 0.52 | 0.65 | 0.46 |
|  | 1.72 | 1.60 | 0.80 | 1.07 |  | 0.47 | 0.80 | 0.40 | 1.21 | 0.66 | 0.67 | 0.53 | 0.66 | 0.47 |
|  | 1.76 | 1.69 | 0.88 | 1.15 |  | 0.53 | 0.81 | 0.40 | 1.25 | 0.66 | 0.71 | 0.53 | 0.70 | 0.50 |
|  | 1.79 | 1.73 | 0.97 | 1.16 |  | 0.54 | 0.81 | 0.42 | 1.53 | 0.67 | 0.72 | 0.54 | 0.73 | 0.50 |
|  | 2.09 | 1.78 | 0.99 | 1.17 |  | 0.54 | 0.84 | 0.43 | 1.62 | 0.67 | 0.72 | 0.56 | 0.74 | 0.51 |
|  | 2.24 | 1.86 | 1.06 | 1.21 |  | 0.57 | 0.85 | 0.44 |  | 0.69 | 0.75 | 0.57 | 0.75 | 0.51 |
|  |  | 2.30 | 1.26 | 1.31 |  | 0.61 | 0.95 | 0.53 |  | 0.85 | 0.79 | 0.65 |  | 0.56 |
| **CENH3 signal intensities** | 2.33e7 | 4.20e7 | 1.90e7 | 3.92e7 | 2.01e7 | 2.60e7 | 6.00e7 | 2.19e11 | 6.14e7 | 7.44e11 | 2.61e7 | 2.50e7 | 1.85e7 | 1.90e7 |
|  | 2.76e7 | 8.26e7 | 2.31e7 | 6.60e7 | 2.16e7 | 2.66e7 | 6.05e7 | 2.50e11 | 6.25e7 | 8.94e11 | 3.93e7 | 3.13e7 | 2.55e7 | 2.21e7 |
|  | 2.79e7 | 8.38e7 | 3.20e7 | 6.97e7 | 2.42e7 | 3.02e7 | 6.24e7 | 2.71e11 | 6.64e7 | 1.30e12 | 4.47e7 | 3.26e7 | 3.49e7 | 2.80e7 |
|  | 3.90e7 | 9.62e7 | 3.59e7 | 7.03e7 | 2.58e7 | 3.15e7 | 6.61e7 | 2.79e11 | 7.33e7 | 1.33e12 | 4.69e7 | 3.41e7 | 3.71e7 | 3.31e7 |
|  | 4.05e7 | 1.06e8 | 3.91e7 | 8.37e7 | 2.78e7 | 3.23e7 | 6.65e7 | 2.87e11 | 7.38e7 | 1.35e12 | 4.78e7 | 3.54e7 | 3.88e7 | 3.32e7 |
|  | 4.06e7 | 1.10e8 | 4.05e7 | 8.77e7 | 3.79e7 | 3.31e7 | 7.26e7 | 3.29e11 | 7.96e7 | 1.36e12 | 5.15e7 | 3.72e7 | 4.06e7 | 3.32e7 |
|  | 4.30e7 | 1.11e8 | 5.22e7 | 9.06e7 | 4.01e7 | 3.50e7 | 7.34e7 | 3.41e11 | 7.98e7 | 1.42e12 | 5.18e7 | 4.04e7 | 4.36e7 | 3.69e7 |
|  | 4.44e7 | 1.30e8 | 5.71e7 | 9.45e7 | 4.23e7 | 3.70e7 | 7.64e7 | 4.08e11 | 8.74e7 | 1.47e12 | 5.18e7 | 4.11e7 | 5.34e7 | 3.78e7 |
|  | 4.82e7 | 1.30e8 | 5.81e7 | 9.76e7 | 6.89e7 | 3.77e7 | 7.68e7 | 4.32e11 | 8.85e7 | 1.48e12 | 5.36e7 | 4.28e7 | 5.39e7 | 3.79e7 |
|  | 5.01e7 | 1.31e8 | 8.23e7 | 9.99e7 | 7.30e7 | 3.82e7 | 8.09e7 | 4.40e11 | 9.08e7 | 1.59e12 | 5.65e7 | 4.40e7 | 5.45e7 | 4.23e7 |
|  | 6.07e7 | 1.37e8 | 6.50e7 | 1.0938 |  | 4.01e7 | 8.22e7 | 4.92e11 | 9.55e7 | 1.67e12 | 5.69e7 | 4.46e7 | 5.48e7 | 4.41e7 |
|  | 6.69e7 | 1.39e8 | 6.52e7 | 1.10e8 |  | 4.20e7 | 8.68e7 | 6.81e11 | 1.19e8 | 1.78e12 | 5.96e7 | 4.49e7 | 5.73e7 | 4.42e7 |
|  | 6.79e7 | 1.40e8 | 7.33e7 | 1.18e8 |  | 4.26e7 | 8.69e7 | 7.06e11 | 1.33e8 | 1.91e12 | 5.98e7 | 4.74e7 | 6.27e7 | 4.47e7 |
|  | 7.36e7 | 1.40e8 | 7.90e7 | 1.22e8 |  | 4.38e7 | 9.04e7 | 7.48e11 | 1.37e8 | 1.94e12 | 6.00e7 | 4.75e7 | 6.74e7 | 4.57e7 |
|  | 9.03e7 | 1.62e8 | 1.01e8 | 1.34e8 |  | 4.84e7 | 1.01e8 | 8.58e11 |  | 2.04e12 | 6.00e7 | 5.96e7 | 6.83e7 | 4.70e7 |
|  |  | 2.70e8 | 1.09e8 | 1.35e8 |  | 4.88e7 | 1.12e8 | 8.73e11 |  | 3.20e12 | 6.23e7 | 6.10e7 |  | 6.00e7 |
